# Efficacy of three surgical methods for gingivectomy of permanent anterior teeth with delayed tooth eruption in children

**DOI:** 10.1186/s13005-022-00328-z

**Published:** 2022-07-07

**Authors:** Dan Xu, Peipei Wang, Hualian Liu, Min Gu

**Affiliations:** Department of Dentistry, the Third Affiliated Hospital of Soochow University, the First People’s Hospital of Changzhou, 185 Juqian Road, Changzhou City, Jiangsu Province 213000 People’s Republic of China

**Keywords:** Laser surgery, Retarded teeth eruption, Gingivectomy, Electrosurgery, Routine surgery

## Abstract

**Objective:**

To compare the efficacy of three surgically assisted permanent anterior tooth eruption methods (laser surgery, electrosurgery and routine surgery) in children.

**Method:**

Sixty-three orthodontic children with retarded permanent anterior tooth were selected and according to the random number table divided into three groups: laser surgery group (group A), electrosurgery group (group B) and routine surgery group (group C). The total operative time (min), the duration of pain after gingival excision (d), Visual Analogue Scale (VAS) pain intensity scores (0–10 cm), and gingival healing time (d) were all recorded. Six months after treatment, periodontal indexes of the three groups, including gingival indexes (GI), plaque indexes (PLI), probing depth (PD) were checked by the same periodontist and recorded.

**Results:**

Surgical records showed that compared with group C, there were statistically significant differences in operative time, pain duration, pain intensity and healing time in group A and B (*P* < 0.05). There was no significant difference in these four results between group A and group B. Periodontal examination indicators 6 months after surgery showed no statistical differences in GI, PLI and PD among group A, B and C. Oral clinical examination found that the three groups of patients with different treatment, dental eruption was normal.

**Conclusion:**

All the three treatments can effectively solve the problem of delayed eruption of permanent anterior teeth in children. Particularly, laser surgery and high-frequency electrosurgery have good efficacy, little pain and high operability, which can be considered as a better method to aid teeth eruption.

## Introduction

The maxillary incisor teeth usually erupt in the early mixed dentition but eruption disturbances can occur and are often attributable to local factors [1]. Early deciduous tooth loss is an important cause of late anterior teeth eruption. During the dentition transitional period, delayed permanent teeth will cause irregular dentition, malocclusion, etc., and it is easy to form cystic changes of surrounding bone, which will cause serious adverse effects on the function, beauty and psychology of children. Therefore, if the permanent incisor is blocked by the tough gingival tissue due to the premature loss of the primary incisor, the opening of the window should be performed as soon as possible [2].

In the process of orthodontic treatment, the method of removing the resistance and exposing the crown is called fenestration. By attaching a hook (bracket) to the tooth surface at the exposed "window" and using the orthodontic method to traction the impacted tooth, the tooth can slowly return to the normal dental arch, restoring the dentition function and the appearance of the tooth [3]. In routine surgery, surgical blade is used to cut tissue directly, which cannot stop bleeding at the same time, often causes more bleeding. Electrosurgical are also commonly used to help the eruption of permanent teeth [4]. Surgical needles and electrodes are very small in size and are easy to use in a confined space in the mouth [5]. High frequency electrosurgical equipment cuts tissue and clots blood by heating and cauterizing. Laser works on different tissue sites by changing the power, output power, pulse frequency and pulse width. New and minimally invasive, laser surgery has a wide range of applications in clinical oral treatment [6], with a good hemostatic effect and a short healing time [7, 8]. Therefore, laser surgery is being used to promote tooth eruption [2, 9, 10]. However, there are few studies on how to better choose one of the three surgical methods for children with delayed permanent anterior teeth. In order to provide effective and valuable evidence in decision making to better select the treatment for permanent anterior teeth in children, this study was designed to compare the efficacy of three surgically assisted permanent anterior tooth eruption methods used in clinical practice.

## Material and methods

### Participants

**Fig. 1:**
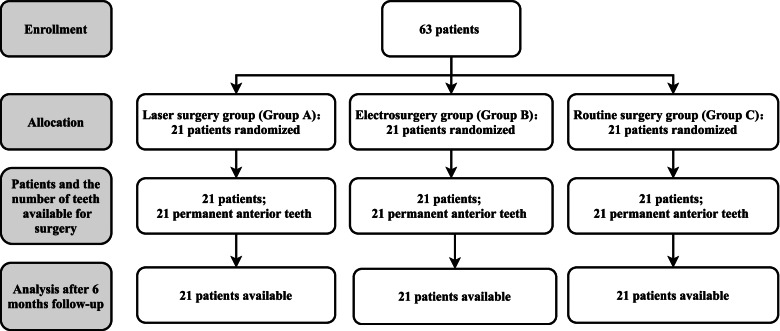
CONSORT flow diagram of the study

A total of 63 patients (age:7–13, gender: 30 boys and 33 girls), who received orthodontic treatment at the Affiliated Third Hospital of Soochow University, Department of Stomatology of Changzhou First People’s Hospital, were recruited from February 2015 to June 2017. The patients were divided into three groups according to the block randomization: ND: YAG laser surgery group (group A), electrosurgery group (group B) and routine surgery group (group C) (Fig. 1).

The following selection criteria were applied: (1) Each subject had only one impacted tooth in the maxillary anterior region; (2) Blood clotting time and other blood profiles were normal before treatment; (3) The delayed eruption of permanent teeth was caused by hypertrophy of the gingiva; (4) The gap left by the missing teeth was sufficient for the eruption of a permanent anterior tooth; (5) Cases showing other causes that impeded the eruption of the teeth, such as supernumerary teeth, root malformation, or cysts, were excluded.

This study involving human participants was reviewed and approved by the First People’s Hospital of Changzhou, the third Affiliated Hospital of Soochow University (202,059). Written informed consent to participant in this study was provided by the participant’s legal guardian next of kin.

### Surgery

Orthodontic treatment runs through the whole process of aid eruption treatment. The main purpose of gingival incision is to eliminate the soft tissue resistance during incisor eruption and open the eruption passage.

#### Preoperative preparation

All surgeries were performed by the same experienced maxillofacial surgeon. All participants underwent curved computed tomography to determine the location and eruption direction of their permanent teeth. When necessary, cone-beam computed tomography (CBCT) was used to diagnose the dental axial direction.

#### Surgical method

All patients were anesthetized with local infiltration of lidocaine. According to preoperative measurement and clinical palpation in oral cavity, the location of surgical incision was determined, and the subgingival crown and buccal surface of late eruption teeth were exposed in three groups by different gingival resection methods.

Group A received a laser ablation of hypertrophic gingiva (Device: LightWalker, brand: Fotona, model: M021-5AF/1, Nd: pulse width: SP; Pulse frequency: 50 Hz; Average output power: 4.5 W) to expose the impacted crown. Laser hemostasis (Nd: pulse width: VLP; pulse frequency: 20 Hz; average output power: 4.00 W) was used to control bleeding. In group B, the hypertrophic gingival was excised with electric knife and cauterized to stop bleeding. Group C received the traditional surgical removal of gingiva with 11 blade. The total operative time (minute), the duration of pain after gingival excision (day), VAS pain intensity scores (centimeter), gingival healing time (day), and intraoperative coordination were all recorded by one trained physician. Pain intensity was assessed using a Visual Analogue Scale (VAS) score (0 to 10 cm), with a VAS marker of 0-2.50 cm for mild pain; 2.60-5.00 cm for moderate pain; 5.10-7.50 cm for severe pain; 7.60-10.0 cm for unbearable pain [10–12].

#### Postoperative management

After the gingiva covering the surface of impacted tooth was removed, cotton balls were used selectively to stop bleeding according to the bleeding situation. No stitches required. Patients in the three groups were washed with normal saline to clean the wound surface. Air gun were used to blow dry tooth surfaces with oil-free air. After the crown surface was dried, the same orthodontist used adhesive (3 M Unitek Transbond™ XT) to bond the orthodontic metal bracket on the exposed surface of the teeth. The traction device was used to connect with the main arch wire and traction was carried out with light force (30-60 g). Patients are advised to maintain good oral hygiene. The patient was followed up by telephone 2 days after surgery, and the persistence of pain was recorded. The gingival healing was observed one week after operation. Six months after the operation, all patients were examined by the same periodontist who was blind to the grouping, and the periodontal indicators such as GI, PLI and PD of the permanent teeth were recorded.

### Detection sites

The mesial, distal, and middle of the labial surface of the permanent anterior tooth after the orthodontic forced eruption.

### Periodontal indexes

The GI [13] was scored as follows: 0 = normal gingiva, 1 = mild edema, no bleeding on probing (BOP), 2 = hyperemia and edema, bleeding on probing (BOP), 3 = obvious redness and swelling, automatic bleeding tendency. PLI [13] was scored as follows: 0 = no plaque on gingival margin, 1 = invisible plaque at the gingival margin but the probe could scrape, 2 = a moderate amount of plaque at the gingival margin or adjacent surface, 3 = a large amount of food debris at gingival crevice or gingival margin and adjacent surface. PD [14] referred to the distance from the gingival margin to the bottom of the periodontal pocket or the bottom of the gingival crevice.

### Data processing

Each periodontal detection site was measured three times and the results of each index were averaged as the final periodontal index of this patient.

### Statistical method

Statistical analysis was performed using SPSS 20.0 statistical software. One-way ANOVA was used for data comparison in groups. *P* < 0.05 was considered statistically significant.

## Results

There was a significant difference in total operative time, pain duration, pain intensity, and healing time between electrosurgery group (group B) and routine surgery group (group C) (*P* < 0.05). And there was also a significant difference in total operative time, pain duration, pain intensity, and healing time between laser surgery group (group A) and routine surgery group (group C) (*P* < 0.05) (Table 1).

**Table 1:** Surgery time, pain duration, pain intensity, healing time for laser surgery group (group A), electrosurgery group (group B), and routine surgery group (group C)

	Surgery time (min)	Pain duration (d)	Visual Analogue Scale (VAS) pain intensity scores (0 - 10 cm)	Healing time (d)
Group A	15.7 ± 2.4^a^	1.5 ± 0.51^a^	2.07 ± 0.58^a^	8.2 ± 1.4^a^
Group B	16.8 ± 2.1^a^	1.59 ± 0.6^a^	2.19 ± 0.69^a^	9.7 ± 0.4^a^
Group C	32.5 ± 3.2	4.4 ± 0.3	5.80 ± 0.57	7.0 ± 0.4

^a^Compared with the group C, *P* < 0.05

Table 2 showed that there were not significant differences among laser surgery group (group A), electrosurgery group (group B), and routine surgery group (group C) in GI, PLI, PD of eruptive permanent teeth after six months of treatment (*P* > 0.05).

**Table 2:** Periodontal indexes of erupted permanent teeth at six months after treatment

	Gingival Indexes (GI)	Plaque Indexes (PLI)	Probing Depth (PD/mm)
Group A	0.98 ± 0.35	1.02 ± 0.52	2.45 ± 0.58
Group B	0.97 ± 0.25	1.10 ± 0.55	2.44 ± 0.62
Group C	0.96 ± 0.65	1.08 ± 0.45	2.46 ± 0.32

## Discussion

Researchers investigated the incidence and severity of impacted teeth with fenestration or fenestration at several dental facilities across Japan. The online survey found that the anterior maxillary region had the highest number of impacted teeth, mostly in the canine region [15]. The eruption of permanent teeth is often delayed when gingival tissue thickens due to premature loss of deciduous tooth and trauma. With effective correction, the teeth can erupt at the normal position, thus reducing the occurrence of malocclusion [11, 12, 16, 17]. Forced eruption can preserve the natural root system and related periodontal architecture, resulting in years of additional service for the patient [18]. The success of surgical exposure combined with orthodontic traction had been reported to exceed 90% [1].

The purpose of this study was to evaluate the efficacy of three surgical methods for gingivectomy of permanent anterior teeth with delayed tooth eruption in children with regard to (1) surgical records and intraoperative pain: the total operative time (min), the duration of pain after gingival excision (d), VAS pain intensity scores (0 to 10 cm), gingival healing time (d) and (2) Postoperative periodontal indicators: gingival indexes (GI), plaque indexes (PLI), probing depth (PD).

The clotting mode of the electric knife can be used to close the blood vessels at the end of the wound, which can not only eliminate intraoperative bleeding, but also provide a good view of the surgical area without suturing [19]. An experimental study by Wang et al*.* showed that 45 children who underwent routine surgery or electrosurgical resection of gingival slices all burst out normally, and the gingiva adhesion and periodontal conditions were good after the eruption of teeth. However, there were statistically significant differences (*P* < 0.01) in bleeding time, operation time, bleeding amount and cooperation of children after gingival resection by electric knife, indicating that high frequency electrosurgical incision is superior to traditional surgical incision [4]. Electrosurgery provides homeostasis by coagulation, seals the capillary and lymphatic vessels, and permits an adequate contouring of the soft tissues. However, high frequency electrosurgical equipment cuts tissue and clots blood by heating and cauterizing, in the use of the electric knife, the paste flavor produced by cauterizing may discomfort the children, make them less cooperative, and prolong the procedures. As an inherent problem in electrosurgery, the use of high-speed evacuators near the operating area can reduce the odor produced [20].

Lasers are being widely used in oral surgery. And laser soft tissue surgery has become gradually accepted in children [21, 22]. Goldman et al. reported the first laser-assisted oral surgery in 1964 [14]. Sarver and Yanosky had summarized that the soft-tissue laser result in a shorter operative time and faster postoperative recuperation [23]. Treated with low-level laser therapy (LLLT) [24], children feel less pain, bleeding, and fear, and are more cooperative. The Nd: YAG laser used in this study emits a laser of 1064 nm to penetrate into the gingiva by a moderate depth, bring much less thermal damage and anxiety to the children [17, 25–28]. Mingwei Li [29] also found that low-level laser can effectively reduce the pain associated with surgical treatment. The result of Li’s experiment is also consistent with this study. This study found a significant difference in total operative time, pain duration, pain intensity, and healing time between laser surgery group (group A) and routine surgery group (group C) (*P* < 0.05). Laser surgery can shorten operative duration, simplify surgical procedures, and reduce postoperative pain and operation-induced fear, all making it highly applicable to children patients [30]. Of course, laser surgery also has obvious disadvantages, such as expense of operatory and upkeep. The major concerns in laser surgery are exposure to laser radiation. Therefore, protective measures must be established, along with proper training of operators and consideration of fire hazards [31].

Final periodontal health is a fundamental key to evaluate the success of therapy for impacted teeth. It is of great advantage to remove periodontal bacteria during the treatment of children with oral hygiene, to prevent gingivitis during orthodontic surgery, and to maintain the periodontal health of patients [32]. Surgical exposure of these impacted teeth is accomplished using various approaches. The appropriate surgical procedure and orthodontic treatment plan will result in a stable, predictable, and aesthetic result [33]. In this study, we did not find significant differences in periodontal indexes among three groups when the permanent teeth emerged at 6 months after the operation. PD values were all within the normal range (PD < 3 mm), meanwhile, no clinical manifestation differences were observed. Some researchers had reported no significant difference in gingival healing between electrosurgery and conventional surgery [34], which was confirmed in this study. This may suggest that although different surgical methods were selected for gingival resection on the crown side of impacted teeth, this factor did not significantly affect the periodontal status after tooth eruption. During normal tooth eruption process, gingival tissue has its own physiological development process [35].

At present, all three methods are clinically applied in gingivectomy of children's permanent anterior teeth to aid eruption, but there are few studies on the evaluation of the three methods. In the study of gingivectomy management of drug-induced gingival overgrowth, some scholars found that scalpel gingival resection and laser surgery had own advantages in plaque scores, bleeding scores, probing pocket depths, pain experience and other aspects respectively [36]. In addition, other studies suggest that electrocautery and laser treatment did not differ significantly in performing gingivectomy procedure and can be used to remove overgrowth of the gingiva with the same efficiency and wound healing power [37]. Even some clinicians have reported greater tactile sense with a scalpel [38]. Given that, it reminds us that the selection of surgical methods for children gingivectomy to aid permanent anterior teeth needs to be considered in many ways.

Pain is defined as “An unpleasant sensory and emotional experience associated with, or resembling that associated with, actual or potential tissue damage” [39]. The first limitation of this study is that the diversity of individuals' pain perception, as well as the external background information they received prior to their participation in the study, can influence patients' pain responses. The complexity and subjectivity of pain that may alter an individual's pain experience, such as fear, may be particularly important considerations [40]. As a special treatment group, children's subjective treatment experience is a factor that doctors should pay attention to when making decisions. Thus, the Kolcaba comfort-behavior scale could be incorporated into the study design in the future.

In addition, there was no significant difference in total operation time, pain duration, pain intensity, healing time, and GI, PLI, PD of eruptive permanent teeth after six months of treatment between the Nd: YAG laser surgery and the high-frequency electrosurgery. Based on the current findings, it is suggested that future studies should focus on evaluating the effectiveness of these two surgical procedures.

## Conclusion

The problem of impacted maxillary anterior teeth is not only an aesthetic dilemma for the dental and maxillofacial regions, but also a problem for social well-being. In view of this situation, Surgical exposure with orthodontic was the most common choice [41]. To find ways to do surgery that would benefit both doctors and patients, this study evaluated the efficacy of laser surgery, electrosurgery and routine surgery for gingivectomy of permanent anterior teeth with delayed tooth eruption in children. In conclusion, the periodontal outcome was not affected distinctly by the operative method. In contrast, the effects of different surgical methods were more obvious during surgery, and the laser surgery group and the electrocautery group had preferable intraoperative performance.

## Data Availability

Data will be available by contacting the corresponding author.

## Abbreviations

VS: Visual Analogue Scale
GI: Gingival indexes
PLI: Plaque indexes
PD: Probing depth
BOP: Bleeding on probing
LLLT: Low-level laser therapy
CBCT: Cone-beam computed tomography
